# Practical Remarks on Epigenesis and Sterility

**Published:** 1858-09

**Authors:** Sam’l A Cartwright


*Practical Remarks on Epigenesis and Sterility.* By SAM'L A CARTWRIGHT, M. D.

The Graafian vesicle is the little bag containing the ovum. It corresponds to the shell of an egg. It is called ovisac. The ovum is one-tenth of a line in diameter. Immediately before orafter menstruation, or its first day, or the last day or two of its continuance, is the time that ninety-five in one hundred conceptions occur. It often takes place immediately before. Then the menses, if they appear at all, soon cease. At these times the ovaria receive more blood, and the ovisacs are inflated and discharge the ova by bursting. The uterus at this time is also supplied with more blood for the formation of the deciduous membrane. There is usually no menstrual flow during pregnancy; it may appear once or twice. The discharge that some women have every month is not menstrual blood properly so called. Before the ovisac or outer covering of the egg or ovum bursts, it becomes vascular in consequence of the determination of blood to the ovaria at the monthly turns, and deposits a fleshy-looking substance of a yellow color. The discharge of ova or little eggs from the ovaries, occurs independent of conception or sexual intercourse, and it is not essential, therefore, that the spermatozoa should reach the ovaries to effect the fertilization of the ova. They may be fertilized while passing to the uterus, through the Fallopian tubes, or in the uterus itself. There are cases proving that they are fertilized while in the ovaria. The farther the ova are extruded the less their capacity for impregnation in the lower animals. But this is not so much the case in the human female. The sexual orgasm is greater during the evolution of the ova at the commencement of catamenia, and a short time after the cessation of the flow. It is only when the ova are set free, that is, burst from the ovisacs or shells, that they are prepared for fecundation. Absolute contact of the spermatozoa with the ovum is essential to fecundation. The spermatazoa are minute particles or germs endowed with self-locomotory powers. They are not animacules, as have been supposed, from their moving rapidly under the microscope. Globules of blood have the same self-locomotory powers. They are particles or germs, male and female, being absolute parts of man's body, parts of the whole man himself, as the bud of the oak is part of the tree, or the bud of the plant, part of a plant. Fecundation is the engrafting of this bud in the ovum, which is a part of the body of the female. Woman's part in the reproduction of the species, is to furnish the stem for the graft or bud, and to supply it with nutriment afterwards.

After the ovum is impregnated, it continues to move downwards to the uterus, and acquires, in its passage through the Fallopian tube, a gelatinous covering, corresponding to the white of an egg. This covering is called the chorion, so called from it being a chorus or company of blood vessels; and from them the graft or bud, transplanted in the ovum, derives its nourishment by means of absorbing radicles, until it arrives in the uterus. By the time it gets there, the deciduous membrane is formed in the uterus to wrap it in, and this membrane is supplied with nourishment to feed it. The villi or little projections or tubes of the chorion are embedded in the fleshy substance of the deciduous membrane and supply nourishment to the embryo, as the homonculus within the ovum is called, after it reaches the uterus.

As the embryo grows and wants more nourishment, another coat is formed over the decidua. This second layer is called the decidua reflexa, to distinguish it for the decidua vera. Little tufts shoot out from the decidua, which imbibe nutriment for the embryo, and ultimately form what is called the after-birth or placenta.

The placenta does not now begin to form until the latter part of the second month. The blood vessels of the uterus are greatly enlarged at the part where the placenta is placed more than elsewhere, and the blood, in moving through the enlarged vessels, produces a peculiar murmur, which is audible at an early stage of pregnancy and is one of the positive signs. It is a cooing sound, like that of blowing over the lip of a wide-mouth vial, always heard in the same spot, but often heard at one time and not at another of the same day or week. It is sometimes heard in the eleventh week, but cannot be relied on before the sixteenth. The mammary gland becomes more developed and the areola of the nipple, and the nipple itself undergo marked changes by the fourth month, being larger and darker. A sense of distension is often felt at the end of the second month, and a tenderness and knottiness occur in the breasts, with occasional shooting pains; but this not an invariable sign. May not happen if the woman be pregnant, and may occur if she is not; which are exceptions, not the rule. A puffy turgescence of the nipple, and an increased size of its little tubercles with a dewy moisture on them, is a more constant concomitant of pregnancy. Sick stomach and excitability of mind attend. Women do not generally know when they conceive, others do, as it produces in them a shuddering sensation, a fluttering movement, attended with dizziness and fainting feelings. Others often feel similar sensations nearly every time they are embraced, and some faint. After conception there is no consciousness of change until quickening. Then a fluttering movement and some degree of syncope are apt to be felt, with a show of a little blood. These sensations are renewed once or twice before the motion of the fœtus is felt by the motion.

Some mothers never feel the motion of the fœtus. The usual or average time of quickening is the eighteenth week. The usual time for parturition is the fortieth week, or two hundred and eighty days. Seldom over that time, but very often a few days under it.

Fecundation does not depend much on the general health of either party, because the generative system has, as it were, a life in some degree independent of the general system, as very sickly looking women often are good nurses, and have healthy children.

The generative system does not come to perfect maturity until after the general system begins to decline. The activity and vigor of perfect manhood, and the freshness, plumpness and beauty of womanhood begin to decline before they have arrived at a stage of life when the powers and capacity of the sexes in reproduction have gained their maximum intensity, which is reached about the 30th year with woman and 40th with man. It is in the beginning of the sear and yellow leaf that the fruit matures, after the flowers have faded The children of very young parents have not the same viability or life-power as the children of parents of maturer age and declining age, nor are they so intelligent. But, after a certain age, about 45 in women, they cease to have children; but up to that time their children are generally more vigorous and intelligent than those they had in early life.

The power of enjoying repeated sexual embraces, declines to less than one-half, before the power of fecundation has arrived at its greatest intensity. Nature appears to have two objects in view in bringing the two sexes together. One, that of love and pleasure, and the other, the perpetuation of the species.

Both are natural instincts, but do not run parallel, and one may exist without the other. In early life, pleasure is uppermost, but in middle life, a desire for offspring is the strongest instinct. Mahometans, and all the Eastern nations, deny women sexual pleasures at any other time than when in a fit condition for conception; after they have conceived, they are set aside and not again embraced until they have had the child and weaned it. The Christian practice is different.

Among Christians, the wife is used as a wife during pregnancy and lactation, as at other times, except the month of confinement, and sometimes the last few days or weeks before that event. During the catamenial period, it is the practice to excuse the wife. Many women are sterile from too strict an interpretation of Moses' laws, which are made to include the evolution of the ova, the first and last days of the vaginal discharge as menstrual, because it is colored, often more deeply than the regular hemorrhagic flow, or menses proper. The lower animals do not mensturate; yet a discharge attends the evolution and bursting of the ova. With them, it is called the pride or heat. In women, it is too apt to be confounded with the catamenia proper, and sexual indulgence prohibited, under a mistaken idea of its being the prohibited flowers of the Old Testament. The prohibition there, could only have been intended to apply to morbid issues and to the menses proper—certainly not to the natural secretions attending ovulation, in a healthy female. Four hundred years before the Christian era, Hippocrates, whose works are still extant and still regarded as the best authority, recommended the period of what is now called ovulation as the proper time for prolific sexual intercourse, viz.: the first and last days of the so-called menstrual discharge, but not during the two or three days of its free and regular flow. He advised fasting on those days to his barren patients, and steaming the uterus with volatile aromatics, by means of an instrument we now call a speculum. To use no bathing or washing, but to wipe off the moisture with dry cloths perfumed, and even to cover up and retain the speculum in its place, and not to remove it until a minute before the embrace is received. Then to lie still, with the limbs crossed, not to make much exertion, or sudden motion of the body, and to live on a plain, and very light diet for five or six days, to prevent the sporangium from perishing and being extruded. If not extruded then, the mouth of the uterus is sealed. There is no danger from inconvenience from subsequent embraces. Twins are supposed to be the product of a second embrace, before the orifice of the uterus has been sealed. It may, however, endanger the first if it is not successful. After impregnation, the fecundating fluid does not reach the cavity of the uterus at all. It either returns as any other foreign substance, or is absorbed. The vaginal walls, in a healthy state, possess great powers of absorption. Medicine can be given that way. When the stomach is irritable and the patient requires cod liver oil, it answers as good, or a better purpose to inject it in the vagina, or introduce a wad of cotton or wool saturated with it. During pregnancy and lactation, and also during the three weeks between the periods, it matters but little whether the fecundating fluid be retained or not.

Some women, about five in one hundred, are impregnated at some time or other of the three weeks from some stray ovum which has been extruded from the ovaries under some strong excitement out of its proper time. Moist and phelgmatic women who cohabit often, had better soak out the fluid with a dry cotton tampon, otherwise health and delicacy require nothing to be done. Many women improperly use injections of cold water or other things. They hurt themselves by injections of an astringent kind, particularly lead, white vitriol, alum, oak-bark, and such substances. They tan the membrane and destroy its natural elasticity. It is a common error to use such injections for fluor albus and for laxity of the parts. The better plan is to encourage the flow by warm mucilaginous applications, to enable the engorged vessels to empty themselves. Then the discharge, an effect of the engorgement, will cease, and the elytron will contract and regain its usual healthy elasticity. Whereas, if the blood in the engorged capillary vessels of the vagina and uterus be brought to a status and fixed in the capillaries by astringents, the parts cannot contract, and the membrane will lose its pliancy and elasticity. The elytron's inner coat is rugose, in some respects resembling the rugosity of the scrotum. When healthy, it is more rugose than in a morbid state. Maladies of a grave kind destroy or nearly obliterate the rugosities of the scrotum. The relaxing and debilitating effects of hot weather act on both those parts pretty much alike. In woman, it is more necessary to keep cool than in the other sex. The practice of wearing drawers, or any thing to heat the parts, is injurious. The thighs and hips are much larger in proportion in woman than any other parts of her body. In proportion to the development of a part, it bears heat less and cold more, because there is more blood distributed to it. There is more blood in that portion of the skin covering the face, and more glands, than in any other portion of the whole surface of the body; and hence the face stands cold better than other parts. The neck and breasts of women are better supplied with glands and capillary vessels than men, and hence they have not the same need of covering. It would be unfortunate if this was not the case, as women would be liable to catch cold from exposing their breasts to nurse their children. The breasts, like the thighs, perineum, and labia majora, should be kept cool. A very large, plethoric woman, with large breasts, had been married some ten years and had no children, though she had had abortions. The treatment advised was, to release her breasts from the flannel garment that encased them and kept them hot enough almost to roast an egg. There was a febrile heat constantly maintained in the uterine region by wearing tight-fitting drawers, which actually excluded the air from the vulva. She was also advised to follow the letter of that portion of Scripture which forbids woman from using man's apparel, or any garment resembling breeches, and consequently to take off her drawers, in obedience to the true original meaning of the Mosaic law, and not fear the cold. She did so, and soon conceived a healthy male child. Long stockings to protect the legs, fastened above the knees, are necessary. Thin pliant shoes are better than bootees. Indian rubber or thick soles are not as healthy as thin, when not walking on damp ground, as they sweat the feet. The thighs should be bare, and the dress should stand off from them. But the body should be kept warm. The breasts should not be pressed upon by the dress so as to be immovable, and the upper part should have air, and be very thinly and loosely covered. Nipples, which have been pressed into the breasts and excluded from the air by a false mode of dressing, become so tender as to bleed and cause the fashionable young mother to scream every time the child's mouth is applied to them. A little learning is a dangerous thing—uneducated women seldom suffer with their breasts during lactation, nor do the highly polished and thoroughly educated. Two-fifths of all the food and drink pass out by the exhalents of the skin. The integuments covering the pudenda, thighs and nates, throw off more effete matter than any other portion of the periphery of the body. If the effete matter does not pass off by the skin, it causes congestions of the womb and other viscera, and is thrown off by a discharge from the mucous membranes, or is exhaled from the lungs, poisoning the breath, or is cast off by the kidneys, causing great irritability in the bladder and inability to retain the urine. Less harm is done when it passes off by the utero-vaginal membrane in the form of what is called whites.' If the latter be dried up by astringents, without removing the cause, the health and complexion of the woman will suffer, and her power of giving and receiving conjugal pleasure will be impaired. The utero-vaginal membrane is best cleansed by its own secretions. Cold water injections fix the matter that ought to pass off. Soap and water immediately relaxes the elytron and destroys the natural acidity of the mucus.

The vulva needs exposure to the external air, as it is the embouchure of a deep current of ga-eous and volatile matters from innumerable conduits, follicles and lacunæ of an extensive number of exhaling organs which have no other outlet. When that is obstructed by tampons or pessaries, by perineal bandages, or any anti-Scriptural mode of dressing, the vaginal secretions not only become highly offensive, but so putrescent, tenacious and acrid as to destroy the vitality of the spermatozoids of the liquor seminis, or greatly to impede their motion. The pubic region not only requires air, but fanning, which is effected in walking, by the flapping of the loose skirts of woman's garments when properly dressed. Hence, sedentary women are less prolific and more subject to uterine diseases and offensive odors, than those who volatilize and dissipate the effete excrementitious humors of the generative system by the fanning exercise in walking. The tenacity and puriform nature of the vaginal secretions, necessarily occasioned by sedentary habits and an improper, unscientific, anti-Scriptural mode of dressing, is a great impediment to the fertilization of the ova, even if those gross acrid humors did not destroy the viability of the zoospermes. It impedes their motions. Their own fluid, the liquor seminis, is too tenacious to admit of their free motion until it is made thinner and lighter by being mixed with a tenuous watery, almost gaseous fluid, secreted by the ovaries and the utero-vaginal follicles, which adds greatly to their motivity. The indelicate and hurtful practice of using cold water injections or astringents under the mistaken idea that they are demanded by cleanliness and give tone to the parts, actually cause what they are intended to prevent, and by astringing the conduits of the follicles, stop up the channels of that thinner and lighter fluid, intended by nature to remove the tenacity of the liquor seminis, and to give activity to the spermatozoa. Women who have abused themselves in this way, or who are afflicted with uterihe maladies, are seldom conscience of any discharge from themselves in sexual embraces. They have little or none, and are not susceptible of any very high degree of pleasurable excitement; while with others it is very abundant, and so light and gaseous as to gush forth under strong excitement like liberated gas or ether It is important to insure the fecundation of an ovum that it meet and commingle at the same instant of time with the liquor seminis containing the zoospermes, to which it imparts a most active motion. That portion of it which comes down from the ovaries washes out the mucosity obstructing the Fallopian tubes and blocking up the mouth of the womb, thereby opening all the conduits to the self-locomotory germs to seek the ova. In a healthy condition of the organs concerned in reproduction, love alone is generally sufficient to insure that sympathy and harmony between the parties so essential to carrying out the designs of nature in perpetuating the species. But there are temperaments so different, and so many functional derangements of the generative system, as utterly to annul the harmonizing influence of the purest conjugal love, unless science be invoked to remove the physical causes obstructing the play of the sexual sympathies between the parties.—*N. O. Med. and Surg. Journal.*

